# Exploring Phylogeographic Congruence in a Continental Island System

**DOI:** 10.3390/insects2030369

**Published:** 2011-08-03

**Authors:** Julia Goldberg, Steven A. Trewick

**Affiliations:** Phoenix Lab, Ecology Group, Institute of Natural Resources, Massey University, Private Bag 11-222, Palmerston North, New Zealand; E-Mail: s.trewick@massey.ac.nz

**Keywords:** New Zealand, Chatham Islands, insects, phylogeographic patterns, mitochondrial DNA, Orthoptera, Coleoptera, Blattodea, Dermaptera

## Abstract

A prediction in phylogeographic studies is that patterns of lineage diversity and timing will be similar within the same landscape under the assumption that these lineages have responded to past environmental changes in comparable ways. Eight invertebrate taxa from four different orders were included in this study of mainland New Zealand and Chatham Islands lineages to explore outcomes of island colonization. These comprised two orthopteran genera, one an endemic forest-dwelling genus of cave weta (Rhaphidophoridae, *Talitropsis*) and the other a grasshopper (Acrididae, *Phaulacridum*) that inhabits open grassland; four genera of Coleoptera including carabid beetles (*Mecodema*), stag beetles (*Geodorcus*), weevils (*Hadramphus*) and clickbeetles (*Amychus*); the widespread earwig genus *Anisolabis* (Dermaptera) that is common on beaches in New Zealand and the Chatham Islands, and an endemic and widespread cockroach genus *Celatoblatta* (Blattodea). Mitochondrial DNA data were used to reconstruct phylogeographic hypotheses to compare among these taxa. Strikingly, despite a maximum age of the Chathams of ∼4 million years there is no concordance among these taxa, in the extent of genetic divergence and partitioning between Chatham and Mainland populations. Some Chatham lineages are represented by insular endemics and others by haplotypes shared with mainland populations. These diverse patterns suggest that combinations of intrinsic (taxon ecology) and extrinsic (extinction and dispersal) factors can result in apparently very different biogeographic outcomes.

## 1. Introduction

A null hypothesis in biogeography is that different taxon groups will show similar patterns of distribution and phylogeny if their evolution has responded to the same historic processes. Some biogeographers have used such a proposal as the basis of a putative test of the role of vicariance in biogeography, under the assumption that patterns associated with dispersal would not be coincident [1–3]. However, early in the application of phylogeographic approaches it was expected that dispersal could also be expected to yield congruent biogeographic patterns, where the taxa involved were responding to a common cause [4] and in many contexts the perceived distinction between vicariance and dispersal processes in biogeography is illusional [5]. In continental systems the past location of species' refugia has been identified using phylogeographic data and found to coincide for many taxa, as does the general trend of expansion from refugia [6,7]. On oceanic islands a general trend in the phylogeographic history of taxa colonizing successive islands as they emerged, has been revealed (e.g., [8,9]). However, discordance is also found and whilst this might be attributed to an overwhelming influence of stochastic events, it might also reflect, at least in part, differences associated with mobility, population size and reproductive strategy as these might influence establishment success [10,11]. Distinguishing between random effects and those linked to species traits is very difficult, especially because traits associated with dispersal might be selected against after colonization [10,12,13].

The study of New Zealand biogeography has in the past focused on the role of plate tectonic vicariance in the origins of New Zealand lineages, but more recently molecular data have shown that many lineages have arrived relatively recently and that diversification is young in many taxon groups [14,15]. Furthermore, it is increasingly evident that there is no uniform pattern in the phylogeographic structuring of the biota within New Zealand [16], with so far, no consistent linkage between lineage formation, landscape history or distribution of taxa being evident. Although New Zealand was influenced by Pleistocene climate cycling, this does not by itself explain the phylogeographic structure of taxa we see today. Not every lineage or population in New Zealand is younger than the last glacial maximum (LGM) [14,16], even though every species and population must have been affected by climate cycling. Although some similarity can be found in lineage ages for different taxa, the pattern of spatial structuring is not consistent among lineages [17]. This was a plausible expectation of species that have similar ecological requirements (e.g., habitat, microclimate, diet) and have experienced range retraction and expansion in a similar time frame (e.g., since the LGM). Instead, what we observe among the extant biota in New Zealand is that some endemic taxa have retained high genetic and taxonomic diversity more consistent with pre-Pleistocene events. On the other hand there are endemic taxa that are widespread with low genetic diversity and in many cases comprising a single mainland New Zealand species (e.g., [18,19]; and see [20]) or a combination of both patterns with widespread species showing low genetic diversity and localized populations/species that retained high genetic diversity within small ranges (e.g., *Phaulacridium* grasshopper [21]; weta, [15,22]). Understanding why different taxon groups have responded so differently requires comparison of taxa across a variety of diverse ecological and evolutionary backgrounds. Their genetic, taxonomic and spatial structuring will help development of more robust hypotheses about the relative role of intrinsic, extrinsic and stochastic processes in the present day biota of New Zealand. Maximising geophysical heterogeneity in such studies allows contrasts to be made between those intrinsic and extrinsic influences and this can be achieved in New Zealand by examining taxa distributed across the mainland and the largest offshore islands, the Chathams.

**Figure 1:**
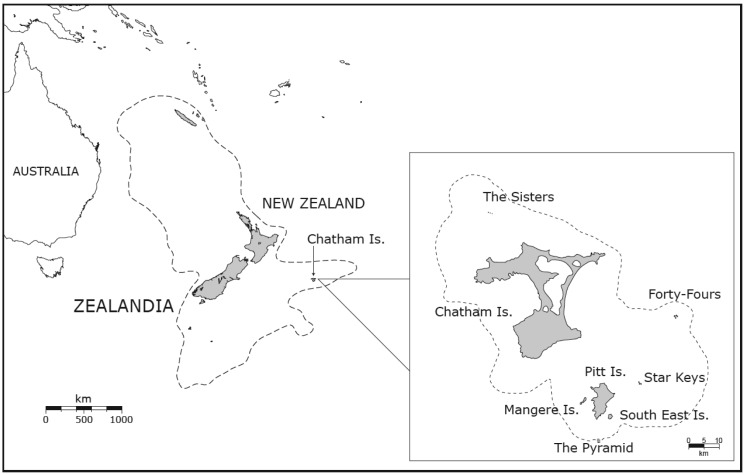
The New Zealand region, showing New Zealand (grey) and the outline of Zealandia (broken line). Inset shows the Chatham Islands (grey) and probable subaerial region during the last glacial maximum (LGM).

The Chatham Islands are located on the Pacific Plate (S 44°03′47.16″ and W 175°57′35.73″) approximately 850 km east of mainland New Zealand (Figure 1) at the eastern end of the Chatham Rise, a submerged ridge-structure extending from mid-South Island. The ridge, together with the Chathams, is part of the same continental crust on which New Zealand is located, and only 10% of the Chathams landmass is above sea level nowadays [23]. The present land above sea level consists of two inhabited islands (Main or Chatham and Pitt Island) and several smaller islets and rock-stacks (Figure 1). Paleomagnetic studies provide evidence that the position of the islands on the eastern tip of the Chatham Rise has been more or less fixed since the break up of Gondwanaland [24]. The geology of the Chathams is unlike most of the other small Pacific island groups because their basement consists of old metamorphic rocks (Chatham schist), similar to the old schists known from the foot of the Southern Alps of Otago and Canterbury, New Zealand. Today the Chatham schist is only exposed in the northern part of the Chathams and on the Forty Fours and its metamorphosis is dated to 160 million years (Myr). On top of this old layer are several younger deposits from the Cretaceous and Cenozoic, which contain the oldest fossils on the Chathams. These fossils indicate a sustained isolation of the Chatham area for the last 65 Myr [24]. In contrast to its settled behavior today, the Chatham area has a long history of volcanic activity, mainly between 60–40 million years ago (Ma) (Eocene to Oligocene) and 5–1.6 Ma (Pliocene). In the latter period most of the smaller islands were formed.

Throughout this history, the Chatham Islands have been submerged and emerged several times in response to regional tectonism affecting the entire eastern end of the Chatham Rise, culminating in the last emergence event no more than 4 million years ago [25,26]. Since then, phases of climate cooling in the Pleistocene (<2 Ma) have influenced the connectivity and land area of the islands [24,27] (Figure 1). Connection of islands during lowered sea level may have enabled population extension and gene flow within the Chathams and mainland New Zealand separately, but could not have influenced New Zealand–Chatham connectivity.

The Chathams therefore provide a convenient context for studying New Zealand biogeography in terms of both spatial (two land areas separated by ∼800 km of ocean) and temporal (emergence ∼4 Ma) scale [28]. Mainland New Zealand is about 270-times larger than the Chathams, and the geographic distance from the mainland to Chathams is about half the length of the main islands (∼1600 km), and 4 Myr is an appropriate time period for exploring species evolution [29]. Furthermore, this Plio/Pleistocene period was one of significant geophysical activity in New Zealand [30]. Thus, there is a nice contrast of highly disjunct *versus* near continuous habitat. Mitochondrial data from taxa in several insect orders present on the Chatham Islands and New Zealand were examined. These data allow assessment of the degree of congruence among genetic diversity, taxonomic status and spatial distribution of mitochondrial lineages within this ecologically diverse set of invertebrate taxa.

## 2. Materials and Methods

**Figure 2:**
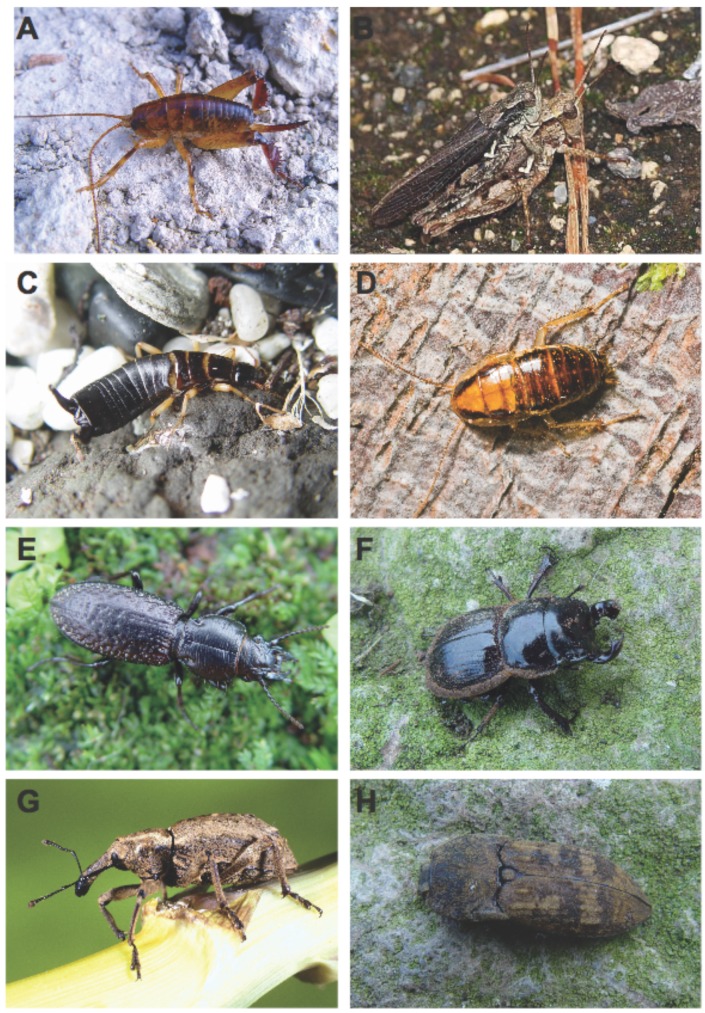
(A) *Talitropsis megatibia*; (B) *Phaulacridium marginale* with rare winged male; (C) *Anisolabis littorea*; (D) *Celatoblatta vulgaris*; (E) *Mecodema crenicole*; (F) *Geodorcus sororum*; (G) *Hadramphus spinipennis*; (H) *Amychus candezei*. Photographs: S. Trewick except B (Mike Lusk), D (Alastair Robertson, Massey University, G (John Marris, Lincoln University).

This study reports on the mtDNA phylogeography of eight New Zealand invertebrate taxa (Figure 2). As such, each component does not assess variation that might exist among genes. While these data represent just one heritable unit within each taxon group, our focus has been on large-scale differences among ecologically diverse taxa. Taxonomic diversity, distribution and abundance of each taxon differs considerably and the scale of analyses varies accordingly, but our sampling reflects approximately their abundance and the strategy was to sample each genus as intensely as possible to include possible sister lineages to the Chatham taxa. The taxa compared share the characteristics of relative large size and flightlessness (with one partial exception), but they differ in their foraging habits, abundance, and landscapes that they occupy. Some of them are subjects of ongoing research, incorporating non-New Zealand outgroup taxa and additional genetic markers, the results of which are in peer-review at the moment and will be published separately elsewhere. Here, we first report data and phylogenetic structure within each genus, we then summarize and compare phylogeographic structure across New Zealand/Chathams.

The invertebrate specimens for this study were collected mainly by hand and preserved in 95% ethanol. They are housed and catalogued in the collection of the Phoenix Lab at Massey University, Palmerston North, with unique voucher numbers. In some cases data were supplemented by specimens from other collections. DNA extraction used leg tissue and a standard salting-out method [31], with whole genomic DNA extractions stored at −20 °C.

The polymerase chain reaction (PCR) was used to target the mitochondrial cytochrome oxidase I (COI) gene region, using in most cases the published oligonucleotide primers C1-J-1718, C1-J2195 and L2-N-3014 [32]. PCR amplifications were performed in a total volume of 10 μL using Red Hot Taq (ABgene). After an initial denaturation at 94 °C for 3 min. DNA was amplified during 35 cycles of 30 s at 94 °C, 45 s at 50 °C and 30 s at 72 °C, followed by a final extension step at 72 °C for 4 min. The PCR cycle conditions varied from above as follows: 35 cycles of 1 min. at 94 °C, 1 min. at 42 °C and 1.5 min. at 72 °C, followed by a final extension step at 72 °C for 5 min. The amplified products were checked on 1% Agarose gels and purified using SAP/EXO1 (USB Corporation) enzyme digest following the manufacturer's instructions. Purified DNA fragments were used for cycle sequencing with Big Dye terminators under standard conditions and read on an ABI 377 sequencer (ABI).

### 2.1. Genetic Analysis

Sequences were edited using Sequencher 4.9 (Gene Codes Corporation) and aligned using SeAl [33]. Mr. Bayes 3.1.2 [34]) was used under a six parameter model selected by jMODELTEST 3.5 [35,36] to reconstruct phylogenetic relationships within each taxon group. MrBayes implemented models incorporating gamma-distributed rate variation across sites and a proportion of invariable sites as appropriate. Four independent MCMC runs for ten million generations with a burn in of 10% were employed for each analysis (results not shown). Unrooted Neigbour-Joining (NJ) networks were also generated for each ingroup dataset of each taxon using HKY distances as implemented in Geneiuos 5.4.3 (Biomatters Ltd.). Uncorrected *p*-distances for all taxa were calculated using PAUP*4 [37]. Where appropriate, DnaSP v5.0 [38] was used to calculate nucleotide diversity (π, [39]), haplotype diversity (*h*) and average number of nucleotide differences (*k*).

### 2.2. Taxa

#### 2.2.1. *Talitropsis* (Orthoptera: Raphidophoridae)-Cave Weta

The family Raphidophoridae (called cave weta in New Zealand) comprises approx. 300 species worldwide [40] with ∼18 genera endemic to New Zealand. *Talitropsis* is a New Zealand endemic consisting of three recognized species, two of which are allopatric endemic species on the Chatham Islands, *T. crassicruris* (Hutton, 1897) and *T. megatibia* Trewick, 1999, and one is widespread throughout mainland New Zealand, *T. sedilloti* Bolivar, 1882. *Talitropsis* is found in forested areas where it hides during the day in holes and cavities and is active at night. On the Chathams, and especially on the smaller islands that lack trees, *Talitropsis* burrows into peaty soil under rocks.

#### 2.2.2. *Phaulacridium* (Orthoptera: Acrididae)-Grasshopper

*Phaulacridium* Brunner v. Wattenwyl, 1893 (Orthoptera: Acrididae) comprises five closely related species, two in New Zealand [*P. marginale* (Walker, 1870) and *P. otagoense* Westerman and Ritchie, 1984], two in Australia [*P. vittatum* (Sjöstedt, 1920) and *P. crassum* Key,1992] and one on Lord Howe Island (*P. howeanum* Key, 1992) [41]. *Phaulacridium* are lowland grasshoppers that inhabit native and mixed exotic herb-/grasslands [41,42]. Most have non-functional reduced wings, but micropterous individuals of *P. vittatum*, *P. crassum* and *P. marginale* do occur [41,42]. *Phaulacridium marginale* is widespread in open grasslands in mainland New Zealand and the Chatham Islands, whereas *Phaulacridium otagoense* is restricted to small semi-arid environments in central Otago and central Canterbury in South Island, New Zealand.

#### 2.2.3. *Anisolabis* (Dermaptera: Labiduridae)-Earwig

*Anisolabis* Fieber 1853 is an earwig genus distributed around the Pacific. However, *Anisolabis littorea* (White, 1846) is an endemic New Zealand species that lives and breeds under rocks and logs on beaches around mainland New Zealand and islands including the Chathams [43]. There are two other species known from New Zealand. *A. kaspar* (Hudson, 1973) is endemic to the Three Kings Islands, northern New Zealand and *A. occidentalis* that has been introduced from Australia, but is restricted to the Hawkes Bay in eastern North Island.

#### 2.2.4. *Celatoblatta* (Blattodea: Blattidae)-Cockroach

The genus *Celatoblatta* Johns 1966 is endemic to the New Zealand region and comprises 13 described species. These nocturnal cockroaches are flightless and species occupy habitats from the subalpine zone to coastal forests. They hide during the day in rotting logs, leaf litter, under bark and rocks. Most of the diversity in this genus is found in South Island, New Zealand and one endemic species (*C. brunni*) is known from the Chatham Islands [44].

#### 2.2.5. *Mecodema* (Coleoptera: Carabidae)-carabid beetle

*Mecodema* (Blanchard, 1843) is a diverse endemic genus of large, flightless carabid beetles (tribe Broscini). *Mecodema* is one of six endemic genera of the tribe Broscini recognized in New Zealand and comprises about 66 species [45,46] and taxa are distributed throughout mainland New Zealand from alpine to coastal habitats. The adults of these beetles are nocturnal, flightless (with fused elytra) and their larvae are predatory [47]. They avoid light and forage within soil and decaying logs for worms and other invertebrate prey. The genus is represented on the Chatham Islands by a population of the species, *M. alternans*.

#### 2.2.6. *Geodorcus* (Coleoptera: Lucanidae)-Stag Beetle

The members of the genus *Geodorcus* are flightless and relatively uncommon. There are 10 described species in *Geodorcus* [48], two of which are endemic to the Chatham Islands, *G. capito* Deyrolle, 1873 and *G. sororum* Holloway, 2007. *Geodorcus* larvae forage on and in decaying logs, but adults sometimes emerge at night onto trees and leaflitter. Most species are now rare and restricted and subject to protection.

#### 2.2.7. *Hadramphus* (Coleoptera: Curculionidae)-Weevil

The endemic weevil genus *Hadramphus* comprises five species with a sparse distribution, including one species restricted to the Chatham Islands (*H. spinipennis* (Broun, 1911)). Taxonomically they belong to the tribe Molytini with the New Zealand genus *Lyperobius* [49]. *Hadramphus* are relatively large flightless weevils (11–23 mm), and both adults and larvae feed on the plants of the families Apiaceae, Araliaceae and one on Pittosporaceae and are therefore restricted to the distribution of these plants. All of the species are endangered and mainly confined to offshore islands or remote areas in Fiordland, South Island. One species, *Hadramphus tuberculatus* (Pascoe, 1877) was rediscovered in Canterbury region during 2004 having last been recorded in 1922 and assumed extinct. *Hadramphus stilbocarpae* (Kuschel, 1971) occurs in Fiordland and Southland, New Zealand, and on the Chatham Islands the endemic *H. spinipennis* feeds and lives on the endemic speargrass *Aciphylla dieffenbachii*.

#### 2.2.8. *Amychus* (Coleoptera: Elateridae)-Click Beetle

*Amychus* are large, flightless click beetles [50]. All species are endangered and are restricted to small predator-free offshore islands. The genus is endemic to New Zealand with three extant species known, including one endemic to the Chatham Islands (*Amychus candezei* Pascoe, 1876). The two other species are restricted to Three Kings Island, (*Amychus manawatawhi* Marris and Johnson, 2010) and islands in Cook Strait (*Amychus granulatus* Broun, 1883). The current distribution suggests that they were probably more abundant and widespread prior to the introduction of mammalian predators, and the populations on Cook Strait islands were connected to one another and to mainland New Zealand as little as 15,000 years ago. *Amychus* generally conceal themselves during the day within and amongst leaf litter and decaying logs or under rocks on peaty soils.

Details of sampling locations and voucher numbers for all taxa employed in this study can be found in the Supplementary Material (Table S1). Details of number of included species per taxon, habitat specification, *etc.*, are listed in Table 1.

## 3. Results

### 3.1. Talitropsis (Orthoptera: Raphidophoridae)-Cave Weta

**Figure 3:**
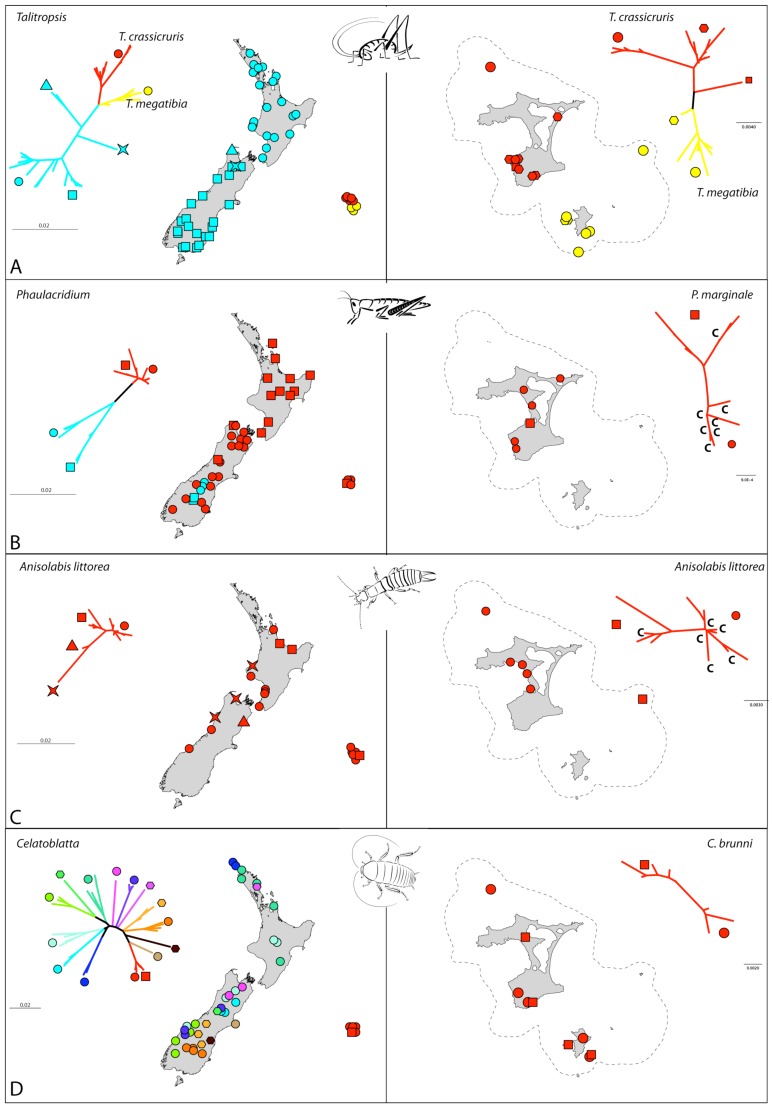

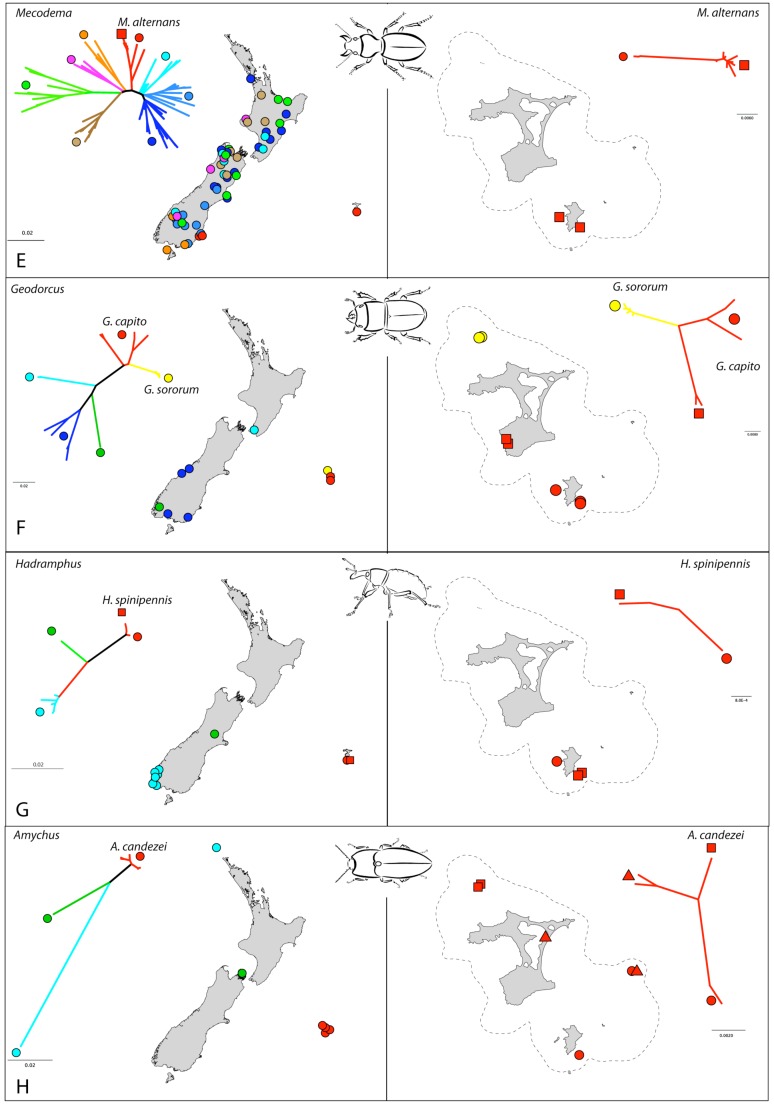
Sampling locations in mainland New Zealand and the Chatham Islands with corresponding unrooted Neigbour-Joining networks. Dashed line indicates likely subaerial Chatham area during LGM. Colored symbols depict different lineages, with colors differentiating between species and symbols between clades. Red and yellow are lineages represented in the Chatham Islands. Where taxa share haplotypes between Chathams and mainland New Zealand, C depicts Chathams sample. (**A**) *Talitropsis* weta. Endemic Chatham species are spatially partitioned. The NZ species *T. sedilotti* is widespread with evidence of recent expansion in North Island (blue circles) and South Island (blue squares); (**B**) *Phaulacridium* grasshopper. Chatham *P. marginale* haplotypes nest among New Zealand haplotypes, and *P. otagoense* (blue) is restricted in mainland New Zealand; (**C**) *Anisolabis littorea* earwig. Haplotypes shared between New Zealand and Chatham; (**D**) *Celatoblatta* cockroaches. Colours indicate species. The endemic Chathams species (*C. brunni* - red) is most closely related to the Banks Peninsula species (*C. peninsularis*– pale brown circle). (**E**) *Mecodema* beetles. Colours indicate species clusters. *Mecodema alternans* is represented in the Chatham by red square, and Dunedin, New Zealand by red circle; (**F**) *Geodorcus* stag beetles. Endemic Chatham species are spatially partitioned but paraphyletic; (**G**) *Hadramphus* weevils. Three endemic species have small, widely spaced ranges. 2H *Amychus* click beetles. Three endemic species have small, widely spaced ranges.

The sampling of *Talitropsis* cave weta included all three recognized species of the genus. The two species on the Chathams are currently isolated from one another with *T. crassicruris* inhabiting Main Island and The Sisters and *T. megatibia* the southern islands of the archipelago including the 44s (Figure 1). The widespread *T. sedilotti* on the other hand occurs throughout the length of mainland New Zealand and several small offshore islands (Figure 2A). A set of 90 specimens comprising all three species of *Talitropsis* were sequenced for a partial fragment (857 bp) of COI (Table S1). Phylogenetic reconstruction of the genus with a suituable outgroup shows distinct monophyletic clades for the species [14]. In *T. sedilotti* there are two additional lineages represented by few individuals from NW South Island, and aside from these the species exhibits some broad regional structuring with samples from North Island and South Islands grouping together but with shallow divergence (Figure 2A and Figure 3A). Within the Chatham species the network splits into two clusters, corresponding to the two endemic species. *T. crassicruris* shows division between the Sisters and Main Island populations, but there is no similar structure apparent among individuals of *T. megatibia* in the southern part of the archipelago. Population genetic statistics calculated for the different species of *Talitropsis* show that nucleotide diversity (π) is similar in the New Zealand species *T. sedilotti* and the Chatham *Talitropsis* together but is lower within each Chatham species (Table 2). Haplotype diversity (*h*) within and between species, on the other hand, is more or less similar. Genetic pairwise distances within *T. sedilotti* were up to 3.12% and almost as high as the distance between the two endemic Chatham species (3.4%). The genetic distance within these two species was up to 2.38% in *T. crassicruris* and 0.98% in *T. megatibia*. Notably there was more genetic diversity between the two taxa in the small Chatham area than within the entire extent of *T. sedilotti* on the mainland. Between the Chathams and New Zealand taxa the genetic distances reached 4.67%. Sequences are deposited in Genbank (JN409905 - JN409993).

### 3.2. Phaulacridium (Orthoptera: Acrididae)-grasshopper

In total 76 individuals, including outgroup taxa (Table S1), were sequenced for COI (763 bp). The two New Zealand species form separate clades with outgroup analysis confirming that *P. marginale* samples form a monophyletic cluster [21]. Haplotypes of *P. marginale* from the Chatham Islands are varied and fall throughout this clade. In contrast to the homogenous and shallow pattern in *P. marginale* throughout New Zealand mainland and the Chatham Islands, there is a deep split within *P. otagoense*, dividing the sampling from two adjacent areas in central South Island (Figure 2B and Figure 3B). Population genetic statistics calculated for the different species of *Phaulacridium* showed that nucleotide diversity (π) was much lower in *P. marginale* including the Chatham samples than within *P. oatgoense* (Table 2). The same is apparent for the haplotype diversity (*h*). Genetic pairwise distances within *P. marginale* were up to 1.8% including Chatham specimens. The genetic distance within *P. otagoense* was similar to this, and 3.5% between the two populations in South Island. Sequences are deposited in Genbank (JN409741 - JN409816).

### 3.3. Anisolabis (Dermaptera: Labiduridae)-Earwig

In total 27 specimens of the widespread species *Anisolabis littorea* and its New Zealand sister taxa were sequenced for a fragment (828 bp) of COI. For *A. kaspar*, unique primer pairs targeting short overlapping sections of COI were designed for the scarce museum material (Table S2) and phylogenetic reconstruction including this species placed it as sister to *A. littorea* [21]. Chatham Island *A. littorea* haplotypes did not form a monophyletic cluster (Figure 2C and Figure 3C), and several haplotypes present in the Chathams had their closest relatives scattered through mainland New Zealand. Genetic distances within the Chatham Island samples were up to 1.2%, and up to 2.9% among all mainland New Zealand and Chathams specimens. Although restricted to a narrow coastal environment this species appears to have had extensive gene flow around New Zealand including the Chathams. This might be due to ongoing exchange or a recent wave of migration involving numerous individuals. Sequences are deposited in Genbank (JN409619 - JN409644).

### 3.4. Celatoblatta (Blattodea: Blattidae)-Cockroach

For *Celatoblatta*, mitochondrial COI (611 bp) was sequenced for 52 specimens comprising 11 species. The Chatham *C. brunni* is sister to *C. peninsularis*, which occupies habitat on Banks Peninsula, the nearest point on the New Zealand mainland to the Chatham Islands [51] (Figure 2D and Figure 3D). Genetic distances between these two species reach a maximum of 3.7%. Some *Celatoblatta* species, in particular in South Island, show strong allopatry to distinct mountain ranges, but other species occupying forest habitats have wider, sometimes overlapping ranges. Sequences are deposited in Genbank (JN409645 - JN409696).

### 3.5. Mecodema (Coleoptera: Carabidae)-Carabid Beetle

Sampling comprised 89 *Mecodema* specimens, representing 37 described and 4 undescribed species (I. Townsend, pers. comm.). COI (788 bp) sequences were obtained, where necessary using specific PCR primers designed to target short overlapping fragments [21]. Bayesian analysis showed strong structure among species groups within *Mecodema* confirming that Chatham *M. alternans* are sister to mainland *M. alternans* in Dunedin [21] (Figure 2E and Figure 3E). In New Zealand, neither species nor species groups are entirely allopatric, suggesting extensive range shifting following speciation. In the case of *M. alternans*, range expansion has included dispersal to the Chatham Islands, although the distribution of the species there is limited. Genetic distances between New Zealand and Chatham Island specimens of *M. alternans* reached 2.9%. Sequences are deposited in Genbank (JN409817 - JN409904).

### 3.6. Geodorcus (Coleoptera: Lucanidae)-Stag Beetle

Mitochondrial COI (777 bp) was sequenced for 20 individuals representing five species, two of which are endemic to the Chatham Islands. Several other species in this genus from mainland New Zealand are exceptionally rare and localized and were not available for analysis. Bayesian analysis with representatives of the closest sister of *Geodorcus* in New Zealand, *Paralissotes*, confirmed the Chatham samples form a monophyletic group apparently sister to the North Island species in the analysis *G. novaezealandiae* [21]. Among the Chatham samples, *Geodorcus capito* from Main Is., South East Is. and Mangere Is., form two clades, so that this species appears to be paraphyletic with respect to the species from The Sisters (*G. sororum*) (Figure 2F and Figure 3F). The genetic distances were up to 0.96% among *G. sororum* haplotypes and 6.9% among *G. capito* haplotypes with as much as 6.75% between these two endemic species. Distances between Chatham and New Zealand species were up to 14.1%. Sequences are deposited in Genbank (JN409697 - JN409716).

### 3.7. Hadramphus (Coleoptera: Curculionidae)-Weevil

The genus *Hadramphus* comprises four species in New Zealand. Three were available for this study, with a forth restricted to an offshore island in northern New Zealand. *Hadramphus spinipennis* is endemic to the Chatham Islands but only occurs on Mangere and South East Islands, were its host plant (*Aciphylla dieffenbachii*) is established. Partial (816 bp) COI was sequenced for 24 individuals of the three species of *Hadramphus* plus additional six species of its closest sister taxon in New Zealand (*Lyperobius*). Bayesian analysis confirmed distinct monophyletic groups for the genera and species, with comparatively shallow diversity in *Hadramphus* [21]. The Chatham Island *H. spinipennis* is, in our sample, sister to Canterbury, New Zealand *H. tuberculatus* (Figure 2G and Figure 3G). However, within *H. spinipennis* there is a very shallow split between the two populations. Genetic distance within the Chatham species was up to 0.5%, with up to 4% between the New Zealand and Chatham species. Sequences are deposited in Genbank (JN409717 - JN409740).

### 3.8. Amychus (Coleoptera: Elateridae)-Click Beetle

Partial (784 bp) COI was sequenced for 17 individuals of three species of the genus *Amychus*, mostly from the Chatham population. These beetles are extremely rare, especially those associated with mainland New Zealand so only one specimen of the Three Kings Is. species, *A. manawatawhi* and two Cook Strait *A. granulatus* were available for analysis. The Chatham endemic species *Amychus candezei* was collected on four islands within the Chatham archipelago (Figure 2H and Figure 3H). The genetic diversity within the Chatham species was up to 1.2%, with up to 4.7% and 9.6% between *Amychus candezei* and *A. granulatus*, and *A. manawatawhi* respectively. Sequences are deposited in Genbank (JN409602 - JN409618).

## 4. Discussion

One prediction widely made by biogeographers is that distribution patterns and phylogeographic structure of different taxon groups are likely to be congruent where they are the product of the same geophysical history. In the present case, emergence of the Chatham Island archipelago from the southern ocean about 4 Ma might be expected to have yielded a set of Chatham Island endemic taxa that were similarly divergent in terms of genetics and morphology, from neighboring New Zealand populations from which they were derived. Although the signature of Chatham diversity is consistently youthful, in keeping with the young age of the islands [28], we found that similarity of mainland New Zealand and Chatham sister lineages ranged from sharing identical haplotypes (e.g., *Phaulacridium* grasshoppers) to having distinct haplotypes differing by as much as 14.1% (*Geodorcus* beetles). Such results are in keeping with previous studies of taxa that incorporate New Zealand and Chatham Island sampling, where divergence estimates range from populations with identical cpDNA haplotypes in the fern *Asplenium hookerianum* [52], to 2% mtDNA sequence divergence in a skink *Oligosoma nigriplantare nigriplantare* [53], and plants with up to 6.4% sequence divergence [54]. Among these examples are spiders [55], insects (cicada [56]; cockroach [51]; damselfly [57]; stick insect [58]), amphipods [59], isopods [60], and birds (rails [61]; robin [62]; parakeet [63,64]; pigeon [18]). Given the wide taxonomic and ecological diversity represented in these studies, perhaps this wide range in divergence estimates reflects differences in biology rather than history, or a combination of the two.

In reality, there are numerous plausible reasons for the variation observed among studies of different taxa, including different rates of lineage extinction; different times of arrival in the Chatham Islands; differences in effective population size; and different rates of molecular evolution. As far as genetic distance is concerned the sampling effect of lineage extinction will always tend to lengthen the inferred distance to a common ancestor from surviving taxa, and the same can result from accidental failure to include close relatives in analyses [5,14,20]. In *Amychus* beetles, for example, biogeographic inference is likely to be strongly influenced by extinction as their present narrow range appears to be a remnant of former diversity [50]. Similarly, high observed divergence in *Geodorcus* was likely exacerbated by gaps in sampling. Some traction is gained in distinguishing these effects in our study when taxonomy (as a proxy for morphological variation) is taken into account, providing an opportunity for comparison that is not available in the majority of published studies that deal with single taxa. Three parameters together contribute information, taxonomy, genealogy and geography, and we note there is no readily discernable concordance of these traits among the taxon groups we studied. Unlike single taxon studies or those with few Chatham samples, here we are able to demonstrate how different the biogeographic patterns expressed by separate taxon groups are (Figure 3). While spatial population structuring within the Chathams might not be expected for taxa that are not also structured between New Zealand and Chathams (e.g., *Phaulacridium* and *Anisolabis*)**, those that are extensively partitioned into species at the source (NZ) might be expected to show equivalent spatial structure at a finer scale (e.g., *Celatoblatta* and *Mecodema*). The Chatham Islands do contain endemic species and sometimes endemic sister species, but these are not consistently associated with genera that are speciose in New Zealand (e.g., *Geodorcus*, *Talitropsis*). Additionally, there is no consistent geographic relationship between New Zealand and Chatham taxa in each group; New Zealand lineages sister to those in the Chathams are in South Island (*Mecodema*, *Celatoblatta*, *Hadramphus*, *Amychus*) or the North Island (*Geodorcus*)—although this is sensitive to taxon sampling, or in the case of the widespread species (*Anisolabis*, *Phaulacridium*) in both of the main islands of New Zealand.

Given the young geological age for the Chatham Islands (Pliocene) and much younger connection among them (Pleistocene low sea level), the incidence of multiple endemics such as *Talitropsis* is intriguing, especially where this contrasts strongly with the situation of their sibling taxa in New Zealand. This is borne out by the respective distribution of genetic diversity, which in the case of *Talitropsis* is indicative of recent southwards range expansion throughout New Zealand (*i.e.*, less genetic diversity in southern parts of the range), whereas in the Chathams, genetic diversity and morphological traits are tightly partitioned over a narrow spatial scale across 50 km of small islands. This is despite the fact that New Zealand and Chathams would each have existed as continuous landscapes during the LGM. Evidently, spatial and temporal effects are not consistent even among sister lineages, and this suggests a strong influence of stochastic processes.

The observation that land area and age might not be accurate predictors of genetic or taxonomic diversification is significant because these are the very traits that are routinely used in biogeographic analyses [5]. Similarly, the demonstration that not only are taxa with “good” dispersal characteristics (e.g., flying rails [61]; pigeons [18]) and windblown fern spores [52] able to reach the Chathams, but so are taxa that lack obvious dispersal attributes. In our study, the presence of flightless insects in the Chathams that are sister to flightless species in New Zealand shows that such animals are not prevented from oversea dispersal. This suggests that passive mechanisms are influential. It might be relevant, for instance, that all the taxa studied spend a substantial amount of their lives in logs, and logs are known to be transported down rivers and to the sea, and to arrive on beaches having drifted in the ocean sometimes carrying animals and plants with them [65]. Records of logs and other flotsam arriving in the Chatham Islands from New Zealand indicate this passage is frequent and rapid. New Zealand *Anisolabis* earwigs are most readily found in and around drift wood on beaches, stag beetle larvae and pupa live and feed in logs, and *Talitropsis* cave weta of all ages occupy holes in wood. Interestingly, in the Chathams the opportunities for log dwelling are fewer on some islands (Forty Fours and The Sisters) where trees are absent. Here, *Talitropsis* occupy cavities in the peaty soil formed between rocks and *Geodorcus* and *Amychus* must complete their life cycles in the humus-rich soil. If passive dispersal is as influential as it appears, we predict that biotic assembly may be mostly contingent upon opportunities for establishment instead.

From examination of eight insect taxon groups comprising many species we conclude that lack of congruence is likely the result of observing evolution at different stages. *Anisolabis littorea* appears to be in or just completing a wave of expansion around the New Zealand coastline including Chathams. There is no indication from the distribution of genetic diversity, that colonizing the Chathams was any more problematic than expansion along the mainland coast; several separate matrilines have made the 800 km trip successfully. The situation in *Phaulacridium marginale* is similar with many genealogical lineages shared across New Zealand and across 800 km of ocean. However, *Phaulacridium* history in New Zealand is intriguing with an endemic species in South Island that contains, in a small geographic area, more genetic diversity than the widespread *P. marginale*. In *Talitropsis*, morphogenesis matches mtDNA partitioning, but genetic distance is not correlated with geographic distance. *Talitropsis sedilotti* appears to have expanded through New Zealand since arrival of the genus on the Chathams, perhaps following local extinction of prior diversity as indicated by distinct localized New Zealand lineages in our data (Figure 3A).

In contrast to this, we see that *Celatoblatta* has maintained taxonomic diversity in New Zealand that may date from before colonization of the Chathams. Whatever process removed most *Talitropsis* diversity (possibly Pleistocene climate cycling) appears to have left *Celatoblatta* relatively intact. We note that in New Zealand, many cockroach species are allopatric but some, especially those associated with forest, have come to occupy relatively wide overlapping ranges. Perhaps even further along in this process, *Mecodema* reveals a well developed New Zealand radiation, apparently retained over protracted time (since Miocene- [21]). Extensive range shifting resulting in sympatry suggests well developed ecological partitioning of the species concerned. In this case arrival in the Chatham Islands appears to have been relatively late in the diversification of the genus. Thus, colonization and speciation appear to have occurred throughout the short geological lifetime of the Chatham Islands, and extinction in New Zealand has influenced the resulting biogeographic patterns.

## 5. Conclusions

Finding biogeographic congruence among taxa, at small or large geographic distances, with or without obvious habitat distinctions and in a time frame that encompasses substantial geogphysical changes is difficult [5]. For the Chatham Islands, the frailty of biogeographic analysis is starkly evident in the former assertion that biological evidence of taxon distribution in the New Zealand/Chatham system was consistent with a process of ancient vicariance [67], whereas it is now clear that neither geology nor biology support this view or are necessary [28,52,54,68]. Two important conclusions arise from this. The first is that if hypotheses about the influence of the size, age and distribution of land on the partitioning of biodiversity cannot be upheld when data about land history are available, what confidence can there be in inferences made about the biogeography of taxa where such data are missing? Secondly, if such landscape traits are not the most important predictors of phylogeographic and phylogenetic partitioning as appears to be the case, then considerable work is needed to describe ecological and behavioral traits of plants and animals so that tests for their biogeographic influence can be made [10,69]. Furthermore, these data are required to refute the hypothesis that stochasticism is the primary force in biogeography.

Future, more intensive sampling of populations and loci may allow assessment of population size and even rates of molecular evolution operating in this system. Natural history studies will contribute to knowledge of population size, behavior and important traits such as longevity and fecundity; all of which influence underlying population genetics. Quantifying these ecological traits will be an important step in elucidating the extent of stochastic processes including extinction and colonization, which are likely to exert strong influence on biogeographic outcomes.

## Figures and Tables

**Table 1:** Summary of taxa studied: a = number of known species in New Zealand/Chatham Is.; b = number of species endemic to Chatham Is.; c = number of species included in this study; NZ = New Zealand; Ch.Is. = Chatham Islands; Genetic dist. = maximum uncorrected genetic distances between sister taxa in NZ and Ch.Is.

**Order**	**Common name**	**Taxon**	**a**	**b**	**c**	**NZ habitat**	**Ch.Is. habitat**	**Genetic dist. (%)**
Orthoptera	cave cricket	*Talitropsis*	(3/2)	2	3	forest	not specific	4.7
Orthoptera	grasshopper	*Phaulacridium*	(2/1)	0	2	open grassland	open grassland	1.8
Dermaptera	earwig	*Anisolabis*	(3/1)	0	3	coastal	coastal	2.9
Blattodea	cockroach	*Celatoblatta*	(13/1)	1	12	not specific	not specific	3.7
Coleoptera	carabid beetle	*Mecodema*	(66/1)	0	35	not specific	not specific	2.9
Coleoptera	stag beetle	*Geodorcus*	(7/2)	2	5	forest	not specific	14.1
Coleoptera	weevil	*Hadramphus*	(4/1)	1	3	host plant	host plant	4.0
Coleoptera	click beetle	*Amychus*	(3/1)	1	3	forest	not specific	9.6

**Table 2:** DNA variation and haplotype diversity within and between regional samples of *Talitropsis* and *Phaulacridium* in the New Zealand region, with the sample size for each region (n), number of observed haplotypes (*N*_haps_), average number of nucleotide differences (*k*)**, nucleotide diversity (π) and haplotype diversity (*h*). Abbreviations represent region as follows: NZ = mainland New Zealand; ChIs = Chatham Islands; NZ Alex = *P. otagoense* population in Alexandra, Otago; NZ Mack = *P. otagoense* population in Mackenzie, Canterbury.

**area**	**n**	***N*_haps_**	***k***	**π (10^−3^)**	***h***
*T. sedilotti* (NZ)	56	33	8.486	1.28	0.948
*T. crassicruris* (ChIs)	22	17	5.381	0.81	0.974
*T. megatibia* (ChIs)	13	10	4.295	0.65	0.962
total ChIs pop.	35	27	8.356	1.26	0.985

*Ph. marg.* (total pop.)	65	5	0.274	0.62	0.180
*Ph. ota.* (NZ Alex)	7	3	2.095	3.50	0.905
*Ph. ota.* (NZ Mack)	4	5	4.667	7.79	0.833
*Ph. ota.* (NZ total pop.)	11	8	9.800	16.36	0.945

**Table S1:** Taxon samples used in this study with voucher numbers and sampling location.

(a) *Talitropsis*.		
**Sample ID**	**Species**	**Location**
CW 48.1	*T. sedilloti*	N.I.,Hawkes Bay,Mohi Bush
CW 47.1	*T. sedilloti*	N.I.,Hawkes Bay,Hastings,Mohi Bush
Tsed4	*T. sedilloti*	S.I., Dunedin, Frasers Bush
Tsed7	*T. sedilloti*	N.I.,Northland,Whatitiri Scenic Res.
Tsed9	*T. sedilloti*	S.I.,Nelson,Nelson Lakes, Mt. Roberts, Carpark
Tsed24	*T. sedilloti*	S.I.,Te Anau,Rainbow Reach
Tsed26	*T. sedilloti*	S.I.,Catlins,Matai Falls
CW 209.1	*T. sedilloti*	N.I.,Te Urewera,Lake Waikaremoana, Hinerau Walk
CW 211	*T. sedilloti*	N.I.,Waikato,Waitomo Caves,Short Bush Walk
CW 210	*T. sedilloti*	N.I.,BOP,nr. Mangatoi,Otanewainuku Forest,Rimu Tr.
CW 207	*T. sedilloti*	N.I.,Mt Taranaki,East-Taranaki,Patea Track
CW 482.1	*T. sedilloti*	S.I., Southland, Takitimu Ra., Pinchester Bush
CW 482.2	*T. sedilloti*	S.I., Southland, Takitimu Ra., Pinchester Bush
CW 482.3	*T. sedilloti*	S.I., Southland, Takitimu Ra., Pinchester Bush
CW 21	*T. sedilloti*	N.I., Auckland, Waitakere, Opanuku Rd
CW 23	*T. sedilloti*	N.I., Northland,Prescot Rd nr Ruakaka
CW 29	*T. sedilloti*	N.I.,Auckland,Waitakere,Arataki
CW 275	*T. sedilloti*	S.I., Otago, Leith Valley, Dunedin
CW 20	*T. sedilloti*	N.I.,Northland, Hukatere
CW 331	*T. sedilloti*	N.I., Coromandel, Cuvier Island
CW 350	*T. sedilloti*	S.I., Nelson Lakes NP, Lake Rotoroa, loop Track
CW 351	*T. sedilloti*	S.I., Lewis Pass NP, Lake Daniells Track
CW 352	*T. sedilloti*	S.I., Fiordland NP, Te Anau, Kepler Track
CW 353	*T. sedilloti*	S.I., Fiordland NP, Te Anau, Kepler Track
CW 354	*T. sedilloti*	S.I., Otago, Queenstown, Kinloch
CW 355	*T. sedilloti*	S.I., Otago, Catlins Coast, Papatowai
CW 45.1	*T. sedilloti*	N.I.,Te Urewera, L. Waikaremoana, Black Beech Track
CW 276	*T. sedilloti*	S.I., Otago, Hampden, Kurinui
CW 371	*T. sedilloti*	N.I., Wellington, Eastbourne
CW 377	*T. sedilloti*	N.I., Waikato, Te Awamutu, Maungatauturi
CW 423	*T. sedilloti*	N.I., Wanganui
CW 457	*T. sedilloti*	N.I., Lady Alice Island
Tsed21	*T. sedilloti*	S.I.,Invercargill, Otarara Scenic Res.
Tsed22	*T. sedilloti*	S.I.,Central Otago, nr. Beaumont
Tsed23	*T. sedilloti*	S.I.,Westland, Haast River
CW 128	*T. sedilloti*	N.I., Northland
CW 428	*T. sedilloti*	N.I., Wanganui
CW 469	*T. sedilloti*	N.I., Taranaki
CW 499.2	*T. sedilloti*	S.I., Invercargill, Bluff Scenic Res.
CW 499.1	*T. sedilloti*	S.I., Invercargill, Bluff Scenic Res.
CW 356.1	*T. sedilloti*	S.I.,Kahurangi NP, Golden Bay,Start of Heaphy Track
CW 356.3	*T. sedilloti*	S.I.,Kahurangi NP,Golden Bay,Start of Heaphy Track
CW 356.2	*T. sedilloti*	S.I.,Kahurangi NP,Golden Bay,Start of Heaphy Track
CW 209.2	*T. sedilloti*	N.I.,Te Urewera,Lake Waikaremoana, Hinerau Walk
CW 209.3	*T. sedilloti*	N.I.,Te Urewera,Lake Waikaremoana, Hinerau Walk
CW 274	*T. sedilloti*	S.I., Otago, Leith Valley, Dunedin
CW 469.2	*T. sedilloti*	N.I., Taranaki
CW 358.1	*T. sedilloti*	N.I., Levin, Tararua Ra., Makahika Lodge
CW 160	*T. sedilloti*	N.I., Manawatu, Levin, Papaitonga Reserve
CW 5	*T. sedilloti*	S.I.,West Coast, Lake Matheson
CW 191	*T. sedilloti*	S.I., Lake Wakatipu, Te Kere Haka Reserve
CW 469.1	*T. sedilloti*	N.I., Taranaki
CW 208	*T. sedilloti*	N.I.,Manawatu, Pohangina Valley, Camp Rangi Woods
CW 481	*T. sedilloti*	N.I., Coromandel, Cuvier Island
CW 481z	*T. sedilloti*	N.I., Coromandel, Cuvier Island
CW 83	*T.crassicruris*	Ch.Is., Main Is., Tuku Reserve, Taiko Camp
CW 102.1a	*T.crassicruris*	Ch. Is., Main Is., Awatotora
CW 102	*T.crassicruris*	Ch. Is., Main Is., Awatotora
CW 102.2	*T.crassicruris*	Ch. Is., Main Is., Awatotora
CW 102.3	*T.crassicruris*	Ch. Is., Main Is., Awatotora
CW 101	*T.crassicruris*	Ch. Is., Main Is., Awatotora
CW 101.1	*T.crassicruris*	Ch. Is., Main Is., Awatotora
CW 101.2	*T.crassicruris*	Ch. Is., Main Is., Awatotora
CW 104	*T.crassicruris*	Ch. Is., Te Whanga Lagoon, Te Mataroe Bush
CW 216	*T.crassicruris*	Ch. Is.,The Sisters,Middle Sister
CW 216.1	*T.crassicruris*	Ch. Is.,The Sisters,Middle Sister
CW 216.2	*T.crassicruris*	Ch. Is.,The Sisters,Middle Sister
CW 212	*T.crassicruris*	Ch. Is.,Main Is.,Hapupu Reserve
CW 214	*T.crassicruris*	Ch. Is.,Main Is.,Southern Tablelands
CW 214.1	*T.crassicruris*	Ch. Is.,Main Is.,Southern Tablelands
CW 205	*T. megatibia*	Ch. Is.,South East Is.
CW 204	*T. megatibia*	Ch. Is.,South East Is.
CW 357	*T. megatibia*	Ch. Is., The Pyramid, Camp Flat
CW 203.1	*T. megatibia*	Ch. Is.,Mangere Is.,Robin Bush
CW 215	*T.crassicruris*	Ch. Is.,The Sisters,Middle Sister
CW 218	*T.crassicruris*	Ch. Is.,Main Is.,Tuku Reserve
CW 02	*T.crassicruris*	Ch. Is.,Main Is.,Whakamarino
CW 213	*T. megatibia*	Ch. Is.,The 44s
CW 219.1	*T. megatibia*	Ch. Is.,South East Is.
CW 219.2	*T. megatibia*	Ch. Is.,South East Is.
CW 219.3	*T. megatibia*	Ch. Is.,South East Is.
CW 206.1	*T. megatibia*	Ch. Is,Mangere Is.,Robin Bush
CW 206.2	*T. megatibia*	Ch. Is,Mangere Is.,Robin Bush
CW 217.1	*T.crassicruris*	Ch. Is.,The Sisters,Middle Sister
CW 217.3	*T.crassicruris*	Ch. Is.,The Sisters,Middle Sister
CW 217.2	*T.crassicruris*	Ch. Is.,The Sisters,Middle Sister
CW 8	*T. megatibia*	Ch. Is.,South East Is.
CW 7	*T.crassicruris*	Ch. Is.,Main Is.,Whakamarino
CW 13	*T. megatibia*	Ch. Is.,Little Mangere Is.
CW 14	*T. megatibia*	Ch. Is.,Little Mangere Is.

(b) *Phaulacridium*.		
**Sample ID**	**Species**	**Location**
PH02	*P. marginale*	N.I., Taupo, Rangipo Desert
PH03	*P. marginale*	N.I., Wellington, Makara
PH05.1	*P. marginale*	S.I., Marlborough, Pelorus Sound
PH06	*P. marginale*	Ch.I., Henga Scenic Res.
PH06.1	*P. marginale*	Ch.I., Henga Scenic Res.
PH04	*P. marginale*	N.I., Tongariro NP, Rangipo Desert
PH07	*P. marginale*	N.I., Wellington, Mt. Kaukau
PH08	*P. marginale*	Ch.I., Fakey's Quarry
PH09	*P. marginale*	S.I., Able Tasman NP, Awaroa
PH10	*P. marginale*	Ch.I., Tuku Res.
PH11	*P. marginale*	Ch.I., Matarakau
PH12.1	*P. marginale*	N.I., Te Urewera, Lake Waikaremoana
PH13	*P. marginale*	Ch.I., Awatotora
PH31.1	*P. marginale*	S.I., Fiordland, Borland Lodge
PH14.1	*P. marginale*	S.I., Otago, Dunedin, Kurinui Hampden
PH14..2	*P. marginale*	S.I., Otago, Dunedin, Kurinui Hampden
PH17.1	*P. marginale*	Ch.I., Maipito Road
PH18.1	*P. marginale*	Ch.I., Tuku Res., Trapline
PH19.1	*P. marginale*	Ch.I., Tuku Res., Abyssinia Track
PH19.2	*P. marginale*	Ch.I., Tuku Res., Abyssinia Track
PH22.1	*P. marginale*	S.I., Able Tasman NP, Awaroa, Dacha
PH22.2	*P. marginale*	S.I., Able Tasman NP, Awaroa, Dacha
PH22.3	*P. marginale*	S.I., Able Tasman NP, Awaroa, Dacha
PH23.1	*P. marginale*	S.I., Lewis Pass NP, Marble Hill Picnic Area
PH24	*P. marginale*	S.I., Canterbury, Hunters Hills, Mackenzie Pass
PH26	*P. marginale*	S.I., Mt Cook NP, Mt Cook Village
PH27.1	*P. marginale*	S.I., Mt. Cook NP, Mt. Cook Village
PH27.2	*P. marginale*	S.I., Mt. Cook NP, Mt. Cook Village
PH27.3	*P. marginale*	S.I., Mt. Cook NP, Mt. Cook Village
PH28.1	*P. marginale*	S.I., Canterbury, Hunters Hills, Myer's Pass
PH29.1	*P. marginale*	S.I., Canterbury, Hunters Hills, Myer's Pass
PH29.2	*P. marginale*	S.I., Canterbury, Hunters Hills, Myer's Pass
PH32.1	*P. marginale*	S.I., Otago, Queenstown, Coronet Peak Skifield
PH35.1	*P. marginale*	S.I., Canterbury, Cave Stream Scenic Res.
PH36	*P. marginale*	S.I., Canterbury, Arthurs Pass
PH37	*P. marginale*	S.I., Seaward Kaikoura Range, Mt. Fyffe
PH38	*P. marginale*	S.I., Marlborough, Clarence River
PH39.1	*P. marginale*	S.I., Marlborough, Kekerengu, Dee Stream
PH49	*P. marginale*	N.I., BOP, Te Puke
PH50.1	*P. marginale*	N.I., Waikato, Maungatatua
PH52	*P. marginale*	N.I., Manawatu, Levin
PH54.1	*P. marginale*	N.I., Hawkes Bay, SH5
PH55.2	*P. marginale*	N.I., Coromandel, Whitianga
PH55.3	*P. marginale*	N.I., Coromandel, Whitianga
PH57.2	*P. marginale*	S.I., Canterbury, Lake Tekapo, Burkes Pass
PH71.1	*P. marginale*	N.I., Whirinaki Forest, Rata Road
PH71.2	*P. marginale*	N.I., Whirinaki Forest, Rata Road
PH71.3	*P. marginale*	N.I., Whirinaki Forest
PHM1	*P. marginale*	S.I., Marlborough, Mt. Patriarch
PHM2	*P. marginale*	S.I., Nelson, St. Arnaud, Mt. Roberts
PHM3	*P. marginale*	S.I., Seaward Kaikoura Range, Mt. Fyffe
PHM4	*P. marginale*	S.I., Otago, Awakino
PHM5	*P. marginale*	S.I., Marlborough, Mt Lyford
PHM6	*P. marginale*	S.I., Otago, Awakino
PHM7	*P. marginale*	S.I., Otago, Awakino
PHM10	*P. marginale*	S.I., Otago, St. Bathans
PHM13	*P. marginale*	S.I., Canterbury, Old Man Range
PHM16	*P. marginale*	N.I., East Cape, East Island
PHM26	*P. marginale*	N.I., Great Barrier Is, Copper Mine
PHMwell	*P. marginale*	N.I., Wellington, Newlands
PHMco2	*P. marginale*	N.I., Coromandel
PHMco3	*P. marginale*	N.I., Coromandel
PHBurk4	*P. marginale*	S.I., Canterbury, Lake Tekapo, Burks Pass
PHoM11	*P. otagoense*	S.I., Otago, Alexandra, Graveyard Gully
PHoM12	*P. otagoense*	S.I., Otago, Alexandra, Graveyard Gully
PHoM14	*P. otagoense*	S.I., Otago, Alexandra, Manor Burn
PHoM8	*P. otagoense*	S.I., Otago, Alexandra, Conroys Dam
PHoM15	*P. otagoense*	S.I., Otago, Alexandra, Bridge Hill
PHoM15.1	*P. otagoense*	S.I., Otago, Alexandra, Bridge Hill
PHo51	*P. otagoense*	S.I., Mackenzie, Lake Tekapo
PHo59	*P. otagoense*	S.I., Mackenzie, Benmore Range, Twizel
PHo60	*P. otagoense*	S.I., Mackenzie, Simons Pass Hill, Tekapo River
PHoCON1	*P. otagoense*	S.I., Otago, Alexandra, Conroys Dam
PHoMtJohn	*P. otagoense*	S.I., Mackenzie, Lake Tekapo, Mt John

(c) *Anisolabis*		
**Sample ID**	**Species**	**Location**
EW 01	*A. littorea*	N.I.,Wellington, The Sirens Rocks
EW 02	*A. littorea*	Ch. Is.,Main, Long Beach South end
EW 03	*A. littorea*	Ch. Is.,Main, The Crossing
EW 04	*A. littorea*	N.I., Manawatu. Tangimoana
EW 05.1	*A. littorea*	N.I.,Bay of Plenty, Papamoa, Tauranga
EW 09.1	*A. littorea*	S.I., Punakaiki, Pahautane Bay
EW 15	*A. littorea*	N.I.,BOP, Ohope Beach,West End
EW 16	*A. littorea*	N.I.,Coromandel,Whitianga,Buffalo Beach
EW 17	*A. littorea*	N.I.,Taranaki,Ohawe Beach
EW 19.1	*A. littorea*	Ch. Is.,Mangere Is.,Robin Bush
EW 21	*A. littorea*	Ch. Is.,Main,Waitangi,Maipito Rd.
EW 23.1	*A. littorea*	Ch. Is.,Main,Hapupu Reserve
EW 25.1	*A. littorea*	Ch. Is.,Main,Ohira Bay, Basalt Columns
EW 27.1	*A. littorea*	Ch. Is.,The 44's
EW 27.2	*A. littorea*	Ch. Is.,The 44's
EW 28.1	*A. littorea*	Ch. Is.,The Sisters,Middle Sister
EW 28.2	*A. littorea*	Ch. Is.,The Sisters,Middle Sister
EW 30.2	*A. littorea*	S.I., Westland, Haast, Shipwreck Beach
EW 34	*A. littorea*	N.I., Manawatu, Foxton Beach
EW 35	*A. littorea*	S.I., Westland, Hokitika Beach
EW 38	*A. littorea*	N.I., Taranaki, New Plymouth
EW 40	*A. littorea*	N.I., Manawatu, Levin
EW 41.1	*A. littorea*	S.I., Seaward Kaikoura, Marfell Beach
EW 42	*A. littorea*	S.I., Able Tasman, Awaroa
EW 39	*A. kaspar*	N.I., Three Kings Islands
EW 06.1	*A. occidentalis*	N.I.,Hawkes Bay, Ngaruroro River mouth
EW 29.1	*A. occidentalis*	N.I., Hawkes Bay, Ocean Beach

(d) *Celatoblatta*.		
**Sample ID**	**Species**	**Location**
CK 01	*C. brunni*	ChIs, South East Is., Woolshed Bush
CK 02	*C. quinquemaculata*	S.I., Otago, Old Man Range
CK 03	*C. quinquemaculata*	S.I., Otago, Rock&Pillar Range
CK04	*C. subcorticaria*	S.I., West Coast, North of Haast
CK 05	*C. vulgaris*	S.I., West Coast, Okuru River
CK 06	*C. sedilloti*	N.I., Northland, Cape Reinga
CK 07	*C. vulgaris*	S.I., Nelson Lakes, St. Arnaud
CK 08	*C. notialis*	S.I., Southlan, Te Anau
CK 09	*C. montana*	S. I. Canterbury, Arthurs Pass, Fog Peak
CK 10	*C. notialis*	S.I., Riverton, Mores Scenic Res.
CK 11	*C. hesperia*	N.I., Northlan, Kaitai, Herekino Forest
CK 12	*C. vulgaris*	N.I., Lake Taupo, Miller's Rd.
CK 13	*C. notialis*	S.I., West Coast, Haast River
CK 14	*C. quinquemaculata*	S.I., Otago, Rock &Pillar Range
CK 15	*C. notialis*	S.I., West Coast, Lake Matheson
CK 16	*C. undulivitta*	N.I.,Whangarei, Mt. Manaia
CK 17	*C. sedilloti*	N.I., Far North, Lake Wahakari
CK 18	*C. sedilloti*	N.I., Whangarei, Waipapa Stream
CK 19	*C. brunni*	ChIs, South East Is., Woolshed Bush
CK 20	*C. quinquemaculata*	S.I., Harris Saddle, Conical Hill
CK 21	*C. anisoptera*	S.I., Otagao, St. Bathans
CK 21b	*C. quinquemaculata*	S.I., Otago, Cromwell, Nevis Rd.
CK 22	*C. montana*	S.I., Canterbury, Craigeburn, Mt. Ida
CK 23	*C. anisoptera*	S.I., Otago, Ohau, Mt. Sutton
CK 26	*C. montana*	S.I., Marlborough, Mt. Lyford, Ammi Range
CK 27	*C. sp.*	S.I., Neslon, Mt. Patriarch, Rochmond Range
CK 28	*C. vulgaris*	S.I., Nelson, Springs Junction
CK 29	*C. sp.*	S.I., Nelson, Springs Junction
CK 69	*C. sp.*	N.I., Northland, Matai Bay
CK 70	*C. undulivitta*	N.I., Northland, Karikari Pensinsula, Lake Ohia
CK 92.1	*C. brunni*	ChIs, Mangere Is., Robin Bush
CK 93	*C. brunni*	ChIs., South East Is., Woolshed Bush
CK 99.1	*C. brunni*	ChIs., The Sisters, Middle Sister
CK 100.1	*C. brunni*	ChIs., Main Is., Nikau Reserve
CK 100.2	*C. brunni*	ChIs., Main Is., Nikau Reserve
CK 103	*C. brunni*	ChIs., Main Is., Tuku Reserve
CK 114.1	*C. brunni*	ChIs., Main Is., Southern Tablelands
CK 115.1	*C. brunni*	ChIs., Main Is., Southern Tablelands
CK 116	*C. anisoptera*	S.I., Mt. Cook Village
CK 129.1	*C. anisoptera*	S.I., Mt. Cook, Kea Point Track
CK 130	*C. sp.*	S.I., Canterbury, Hunters Hills, Meyer's Pass
CK 131	*C. vulgaris*	N.I., Lake Taupo, Kinloch, Kawakawa Track
CK 132	*C. notialis*	N.I., Fiordland, Te Anau, Kepler Track
CK 133	*C. notialis*	S.I., Fiordland, Milfors Rd., Key Summit
CK 135	*C. subcorticaria*	S.I., Canterbury, Arthurs Pass, Cockayne Nature Walk
CK 138	*C. subcorticaria*	S.I., Otagao, Mt. Aspiring, Makarora
CK 141.1	*C. brunni*	ChIs., Pitt Is., North Head
CK 147	*C. hesperia*	S.I., Canterbury, Arthurs Pass, Cockayne Nature Walk
CK 148.1	*C. undulivitta*	N.I., Wairarapa, Norsewood, ANZAC Reserve
CK 155	*C. undulivitta*	N.I., Coromandel, Kauri Grove, Waiau
CK 158.1	*C. pensinsularis*	S.I., Canterbury, Banks Pensinsula
CK 158.2	*C. pensinsularis*	S.I., Canterbury, Banks Pensinsula

(e) *Mecodema*.		
**Sample ID**	**Species**	**Location**
MB 01	*M. alternans*	Chatham Is., South East Is.
MB 02	*M. alternans*	Chatham Is., South East Is.
MB 70	*M. alternans*	Chatham Is., Mangere Is.
MB 71	*M. alternans*	Chatham Is., South East Is.
MB 86	*M. alternans*	Chatham Is., Mangere Is.
MB 87	*M. alternans*	Chatham Is., South East Is.
MB 14	*M. alternans*	S.I., Dunedin, Taieri Mouth
MB 16	*M. alternans*	S.I., Dunedin, Sandfly Bay
MB 79	*M. crenicolle*	S.I., Marlborough Sounds, Pelorus Bridge
MB 103	*M. crenicolle*	S.I., Nelson Lakes, Wairau River
MB 66	*M. curvidens*	N.I., BOP, Rotorua
MB 110	*M. fulgidum*	S.I., Clarence Valley, Mt. Percival
MB 91	*M.* cf *fulgidum*	S.I., Seaward Kaikoura Ra., Mt. Lyford
MB 98	*M. howittii*	S.I., Canterbury, Banks Peninsula
MB 99	*M. howittii*	S.I., Canterbury, Banks Peninsula
MB 63	*M. longicolle*	N.I., Ruahine Ra., Pohangina Valley
MB 19	*M. lucidum*	S.I., Otago, Carrick Range
MB 11	*M.* nsp.	S.I., Central Otago, Old Man Range
MB 37	M. nsp.	S.I., Arthurs Pass NP, Dome
MB 51.1	M. nsp.	N.I., Hawkes Bay, Havelock North
MB 68	*M. occiputale*	N.I., Mangatoi, Otanewainuku Forest
MB 23	*M.* cf *oconnori*	N.I., Wellington, Levin, Ohou
MB 90	*M. oregoides*	S.I., Christchurch, Akuriri Scenic Res.
MB 03	*M. rugiceps*	S.I., Fiordland, Lake Harris
MB 45	*M. sculpturatum*	S.I., Dunedin, Ross Reserve
MB 108	*M.* cf *huttense*	S.I., Canterbury, Peel Forest
MB 25	*M. simplex*	N.I., Manawatu, Pahiatua Track
MB 64	*M. simplex*	N.I., Manawatu, Palmerston North
MB 50	*M. spinifer*	N.I., Hawkes Bay, Mohi Bush
MB 18	*M. spinifer*	N.I., Auckland, Waitakeres, Arataki
MB 96	*M. strictum*	S.I., Nelson, Takaka Hill, Canaan
MB 95	*M. sulcatum*	S.I., Kaikoura, North of Ohau Point
MB 69	*M. validum*	N.I., Tongariro NP, Whakapapanui Track
MB 86.1	*M. alternans*	Chatham Is., Mangere Is
MB 88	*M. alternans*	Chatham Is., South East Is.
MB 88.1	*M. alternans*	Chatham Is., South East Is.
MB 190	*M. alternans*	S.I. Otago, Takahopa River Mouth
MB185	*M. alternans hudsoni*	The Snares [LUNZ 00002804]
MB 176	*M. rectolineatum*	S.I., Remarkables Range, Wye Creek
MB 196	*M. politanum*	S.I., The Remarkables, Rastus Burn
MB 128	*M. impressum*	S.I., Queenstown, Kinloch
MB 125	*M. sculpturatum*	S.I., Catlins Forest Park, River Walk
MB 111	*M. lucidum*	S.I., Pisa Range
MB 123	*M. fulgidum*	S.I., Hamner Springs, Forest Park,
MB 134	*M. constrictum*	S.I., Craigeburn Forest P., Education Ct.
MB 100	*M. costellum lewisi*	S.I., Road nr. Mt. White Station
MB 101	*M. costellum obesum*	S.I., Nelson, Takaka Hill, Canaan
MB 124	*M. nsp.*	S.I., Lewis Pass, Lewis Tops
MB 195	*M. allani*	S.I., Nelson Lakes, Matakitaki V.
MB 197	*M. laterale*	S. I., Fiordland, Routeburn Track
MB 178	*M. minax*	S.I., Catlins, Mt. Pye Summit
MB 180	*M. minax*	S.I., Catlins, Mt. Pye Summit
MB 160	*M. elongatum*	S.I., Otago, Kinloch
MB 143	*M. metallicum*	S.I., Buller, Fox River
MB 117	*M. crenicolle*	S.I., Lake Rotoroa, Braeburn Walk
MB 163	*M. crenaticolle*	N.I., Taranaki, Kaiteke Ra., Lucy's Gully
MB 186	*M. crenaticolle*	N.I., Waikato, Skyline Cave
MB 121	*M. ducale*	S.I., Lewis Pass, Lake Daniels Walk
MB 147.1	*M. oregoides*	S.I., Canterbury, Ahuriri Scenic Res.
MB 187	*M. undet.*	S.I., Otago, nr Cromwell
MB 30	*M. undet.*	S.I., Able Tasman, Awaroa
MB 38	*M. undet.*	S.I., Takaka, The Grove
MB 49	*M. undet.*	S.I., Takaka, Rameka Track
MB 65	*M. occiputale*	N.I., BOP,Ohope Scenic Res.
MB 67	*M. occiputale*	N.I., Mangatoi,Otanewainuku Forest,
MB 61	*M. crenaticolle*	N.I., Taranaki, Lake Rotokare
MB 62	*M. crenaticolle*	N.I., Taranaki, Lake Rotokare
MB 72	*M. crenaticolle*	N.I., Tongariro, Ohakune
MB 12	*M. crenicolle*	S.I., Nelson, Shenandoah
MB 44	*M. crenicolle*	S.I., Able Tasman, Awaroa
MB 82	*M. crenicolle*	S.I., Able Tasman, Awaroa
MB 54	*M. morio*	S.I., Catlins, Purakaunui Falls
MB 105	*M. infimate*	S.I., Codfish Island
MB 76	*M. simplex*	N.I., Tararua Forest Park, Putara
MB 77	*M. simplex*	N.I., Tararua Ra., Mt Holdsworth
MB 35	*M. constrictum*	S.I., Fog Peak, Porter's Pass
MB 50.1	*M. spinifer*	N.I., Hawkes Bay, Mohi Bush
MB 81	*M. fulgidum*	S.I., Cobb Valley, Lake Sylvester Tr.
MB 27	*M. constrictum*	S.I., Canterbury, Craigeburn Rec. area
MB 07	*M. nsp.*	S.I., Seaward Kaikoura Ra., Mt. Fyffe
MB 20	*M. cf oconnori*	N.I., Levin, 30B The Avenue
MB 73	*M. cf oconnori*	N.I., Te Urewera, Ngamoko Trig Tr.
MB75	*M. cf oconnori*	N.I., Dannevirke, Norsewood Res.
MB 10	*M. spinifer*	N.I., Auckland, Waitakares Ra.
MB 17	*M. punctatum*	S.I., Rock&Pillar Range
MB 04	*M. sculpturatum*	S.I., Dunedin, Leith Saddle
MB 06	*M. sculpturatum*	S.I., Dunedin, Mosgeil, Silver St.
MB 09	*M. huttense*	S.I., Canterbury, Peel Forest
MB 46	*M. cf huttemse*	S.I., Canterbury, Peel Forest

(f) *Geodorcus*		
**Sample ID**	**Species**	**Location**
SB 03	*G. capito*	Ch. Is., South East Is.
SB 06	*G. capito*	Ch. Is., Taiko Camp,Mt. Albert
SB 25	*G. capito*	Ch. Is., South East Is., Woolshed
SB 41	*G. capito*	Ch. Is., Mangere Is., Robin Bush
SB 42	*G. capito*	Ch. Is., South East Is., Woolshed
SB 47	*G. capito*	Ch. Is.,Main Is.,Tuku Reserve
SB 02	*G. sororum*	Ch. Is., The Sisters
SB 24	*G. sororum*	Ch. Is., The Sisters
SB 46	*G. sororum*	Ch. Is.,The Sisters,Middle Sister
SB 46.1	*G. sororum*	Ch. Is.,The Sisters,Middle Sister
SB 46.2	*G. sororum*	Ch. Is.,The Sisters,Middle Sister
SB 54	*G. sororum*	Ch. Is.,The Sisters,Middle Sister
SB 29.1	*G. philpotti*	S.I., Southland, Borland Saddle
SB 27	*G. novaezealandiae*	N.I., Wellington, Catchpole
SB 72.1	*G. novaezealandiae*	N.I., Wellington, Eastbourne, Butterfly Creek
SB 72.2	*G. novaezealandiae*	N.I., Wellington, Eastbourne, Butterfly Creek
SB 01	*G. helmsi*	S.I., West Coast, Copeland Track
SB 04	*G. helmsi*	S.I., Salmon Farm,North of L. Paringa
SB 21	*G. helmsi*	S.I., Southland, Riverton, Mores Scenic Res.
SB 69	*G. helmsi*	S.I., Otago, Catlins Coast, Papatowai

(g) *Hadramphus*.		
**Sample ID**	**Species**	**Location**
Wv 02	*H. stilbocarpae*	S.I.,Fiordland, Breaksea Is. headland
Wv 03	*H. stilbocarpae*	S.I.,Fiordland, Breaksea Is. headland
Wv 04	*H. stilbocarpae*	S.I.,Fiordland, South Breaksea Islet
Wv 05	*H. stilbocarpae*	S.I.,Fiordland, South Breaksea Islet
Wv 06	*H. spinipennis*	Ch. Is.,South East Island II
Wv 07	*H. spinipennis*	Ch. Is.,South East Island II
Wv 08	*H. spinipennis*	Ch. Is., South East Island
Wv 09	*H. spinipennis*	Ch. Is.,Mangere Island patch
Wv 10	*H. spinipennis*	Ch. Is.,Mangere Island patch
Wv 11	*H. spinipennis*	Ch. Is.,South East Island I
Wv 12	*H. spinipennis*	Ch. Is.,South East Island I
Wv 13	*H. spinipennis*	Ch. Is.,Mangere Island patch
Wv 14	*H. spinipennis*	Ch. Is., 28 Keefo van patch 3
Wv 15	*H. spinipennis*	Ch. Is.,Mangere Island patch
Wv 16	*H. spinipennis*	Ch. Is.,Mangere Island patch
Wv 17	*H. spinipennis*	Ch. Is.,South East Island
Wv 23	*H. stilbocarpae*	S.I., Fiordland, Secretary Is.
Wv 24	*H. stilbocarpae*	S.I., Fiordland, Hawea Is.
Wv 25	*H. stilbocarpae*	S.I., Fiordland, Puysegur Point
Wv 26	*H. stilbocarpae*	S.I., Fiordland, Chalky Is.
Wv 27	*H. stilbocarpae*	S.I., Fiordland, Wairaki Is.
Wv 28	*H. stilbocarpae*	S.I.,Fiordland, Breaksea Is.
Wv 18	*H. tuberculatus*	S.I., Canterbury
Wv 31	*H. tuberculatus*	S.I., Canterbury

(h) *Amychus*		
**Sample ID**	**Species**	**Location**
CB 04	*A. manawatawhi*	N.I., Three Kings Is., Great Island, Tasman Va.
CB 14.1	*A. candezei*	Ch. Is., South East Is.,Woolshed Bush
CB 14.a/2	*A. candezei*	Ch. Is., South East Is.,Woolshed Bush
CB 14.3	*A. candezei*	Ch. Is., South East Is.,Woolshed Bush
CB 15	*A. candezei*	Ch. Is.,Main, Hapupu Reserve
CB 16.1	*A. candezei*	Ch. Is.,The 44s
CB 16.1/2	*A. candezei*	Ch. Is.,The 44s
CB 16.a/2	*A. candezei*	Ch. Is.,The 44s
CB 16.3	*A. candezei*	Ch. Is.,The 44s
CB 16.4	*A. candezei*	Ch. Is.,The 44s
CB 17.1	*A. candezei*	Ch. Is.,The Sisters,Middle Sister
CB 17.2	*A. candezei*	Ch. Is.,The Sisters,Middle Sister
CB 17.2a	*A. candezei*	Ch. Is.,The Sisters,Middle Sister
CB 17a	*A. candezei*	Ch. Is.,The Sisters,Middle Sister
CB 17a/2	*A. candezei*	Ch. Is.,The Sisters,Middle Sister
CB 22	*A. granulatus*	S.I.,Cook Strait,Maud Is.,DoC house
CB 23	*A. granulatus*	S.I.,Cook Strait,Maud Is.,DoC house

ChIs. = Chatham Islands; N.I. = North Island, New Zealand; S.I. = South Island, New Zealand.

**Table S2:** Table of primers designed for this study to amplify the COI fragment in *Anisolabis kaspar*, incorporating the IUPAC code.

**Primer name**	**Sequence**	**Gene**
EW_114R	3′ GTAGGTACAGCAATAATT	COI
EW_115F	5′ ATTATTGCTGTACCTACMG	COI
EW_322R	3′ AKACTGCTCCTATAGAAAGAAC	COI
EW_293F	5′ CTTATTATGTTGTWGCTCAC	COI
EW_514R	3′ CAACAWATATAAGCATCAGG	COI
EW_489F	5′ GATACCTCGWCGATAYTCAG	COI
EW_679R	3′ CTATGRTCTGMTGGTGGA	COI
